# Chemical and Thermal Stability of Sr_1.9_VMoO_6−δ_: Implications for High Temperature Energy Conversion Applications

**DOI:** 10.1021/acsomega.5c06796

**Published:** 2026-02-02

**Authors:** Bamidele J. Samuel, Julia A. Esakoff, Stephen K. Heywood, Stephen W. Sofie, Robert A. Walker

**Affiliations:** † Department of Chemistry and Biochemistry, 33052 Montana State University, Bozeman, Montana 59717, United States; ‡ Department of Mechanical and Industrial Engineering, Montana State University, Bozeman, Montana 59717, United States; § Montana Materials Science Program, Montana State University, Bozeman, Montana 59717, United States

## Abstract

Raman spectroscopy and thermal gravimetric analysis (TGA) were used to evaluate the thermal and atmosphere stability of Sr_1.9_VMoO_6−δ_ (SVMO-19), an A-site deficient double perovskite. Motivated by previous reports describing SVMO-19’s unprecedented electrical conductivity under reducing atmospheres, studies described in this work determine SVMO-19’s stability under conditions commonly encountered in high temperature solid oxide electrolysis and fuel cell applications. Vibrational Raman data show that SVMO-19 is stable up to 1000 °C under reducing, inert, and CO_2_ containing atmospheres. Under air, however, *in situ* Raman data show that SVMO-19 phase separates at temperatures ≥ 600 °C. The primary degradation products include a scheelite phase (SrMoO_4_) as well as a vanadium containing single perovskite, SrVO_3_, and a vanadium containing pyrochlore Sr_2_V_2_O_7_. TGA measurements suggest that SVMO decomposition in air begins at even lower temperatures (400 °C). TGA data show that SVMO is stable under N_2_ at temperatures as high as 900 °C, consistent with Raman data. SVMO oxidation kinetics are analyzed using both a simple kinetic model consisting of two independent first-order processes and an Avrami model. The data are better described by the pair of first order processes, but an Arrhenius analysis using both models result in an activation energy (*E*_a_) for SVMO degradation between 0.48 and 0.65 eV. Taken together, these findings are considered in the context of properties required by electrode materials used in reversible solid oxide electrochemical cells.

## Graphical abstract

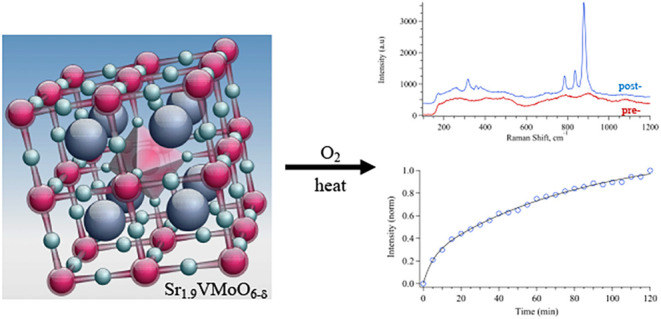

## 1 Introduction

High temperature energy conversion devices such as solid oxide fuel cells (SOFCs) and solid oxide electrolysis cells (SOECs) require electron and ion conducting materials that are stable under chemically aggressive conditions including oxidizing/reducing atmospheres, applied overpotentials, and temperatures approaching 1000 °C. Given these limitations, most traditional SOFCs and SOECs rely on oxide-conducting yttria stabilized zirconia (YSZ) as an electrolyte with a nickel-YSZ cermet serving as a fuel electrode and a mixed ion-electron conducting (MIEC) perovskite as an oxygen electrode.^1,2^ Some variants to this design include ceria-based electrodes and electrolytes as well as porous, electron-conducting ceramics as fuel electrodes.^3^

Fuel electrodes face major challenges to stable, long-term operation in SOFCs and SOECs. Ni-based electrodes, in particular, are susceptible to coking, metal dusting, and sulfur poisoning that can yield irreversible degradation to the anode.^4–10^ If used as an electrode in reversible SOFC/SOEC applications, a Ni-based electrode can be subject to strong oxidizing conditions that lead to NiO formation and significant mechanical stress on device components.^11,12^ To address these issues, efforts have focused on improving resistance to carbon accumulation and sulfur tolerance employing new anode materials such as alloys and conducting ceramics whose catalytic activity is enhanced through infiltration or nanoparticle exsolvation.^13^ These improvements, however, often come at the cost of other key properties, such as electrocatalytic activity, conversion efficiency, long-term stability, and cost.

Perovskite oxides (ABO_3−δ_) are promising candidates for general fuel electrode applications, due to their ability to serve as mixed electronic and ionic conductors (MIECs). By replacing conventional nickel (Ni) and yttria-stabilized zirconia (YSZ) with a perovskite MIEC, the electrode’s electrochemically active region is no longer confined to a narrow triple-phase boundary (TPB) making the electrode less susceptible to contamination and degradation. Furthermore, certain perovskite compounds have proven robust in extreme conditions including high temperature reducing and oxidizing atmospheres,^14^ while also showing exceptional catalytic activity for hydrogen and methane oxidation,^15,16^ sulfur tolerance,^17^ and resistance to carbon deposition.^18^

Double perovskites have the general formula A′A″B′B″O_6−δ_ and afford even greater flexibility over average B-site oxidation state(s) and oxide vacancy concentrations. These materials have shown more promise than single perovskites in terms of their ionic conductivity and resistance to common SOFC/SOEC contaminants.^19–23^ By integrating two different metal cations into the material’s B-sites, double perovskites can accommodate one transition metal in a high oxidation state (*e.g.*, Mo^6+^, Ta^5+^) to enable a higher number of oxide vacancies and a second metal that suppresses A-site species mobility, a common source of perovskite instability.

Double perovskite oxides such as Sr_2_Fe_1.5_Mo_0.5_O_6_ exhibit high oxide ion conductivity, reaching 0.13 S/cm at 800 °C, and have demonstrated promising performance as cathode materials in intermediate-temperature electrochemical devices.^24^ In addition to their ion conducting capabilities, double perovskites can also have appreciable electronic conductivities. Previous studies have reported that certain double perovskite oxides exhibit moderate electronic conductivities under reducing conditions at 800 °C, with values such as 5.3 S/cm for (Sr_2_FeNb_0.2_Mo_0.8_O_6−δ_)^25^ and approximately 1 S/cm for (La_0.75_Sr_0.25_Cr_0.5_Mn_0.5_O_3_).^26^ However, significantly higher conductivities have been achieved through targeted B-site doping, as demonstrated by Sr_2_FeMo_0.6_Mg_0.25_Ga_0.15_O_6−δ_ (36 S/cm)^27^ and Sr_2_Fe_1.5_Mo_0.5_O_6−δ_ (∼17 S/cm).^28^ Notably, the introduction of A-site deficiency has led to a dramatic enhancement in conductivity, with values reaching 1250 S/cm for Sr_2_VMoO_6–*y*′_, 2530 S/cm for Sr_1.8_VMoO_6–*y*″_, and up to 3610 S/cm for Sr_1.9_VMoO_6–*y*′″_ all at 800 °C in 5% H_2_/95% N_2_.^29^

Despite these attractive performance metrics, double perovskite electrodes suffer from material decomposition into secondary phases including single perovskites (*e.g.*, SrMO_3_) and a scheelite phase (*e.g.*, SrMO_4_).^30^ (Here, M corresponds to B-site constituents in the parent material). The scheelite phase is particularly problematic, given that these materials are often both electronically and ionically insulating. Son et al.^31^ used a host of methods including X-ray diffraction (XRD), thermogravimetric analysis (TGA), thermomagnetization (TM) and Raman spectroscopy to show that the double perovskite Sr_2_FeMoO_6_ (SFMO) remained stable in N_2_ at temperatures up to 900 °C, but in air this material began to degrade above 400 °C. *Post-mortem* analyses showed SrFeO_3_ and SrMoO_4_ to be the dominant secondary phases, but SFMO’s degradation kinetics and the mechanisms responsible for decomposition into these secondary phases remained unresolved.

Results reported in this work examine how the double perovskite SVMO-19 behaves under conditions typically encountered in SOFCs and SOECs. This material has been cited as having unusually high electronic conductivities and has shown resilience to sulfur poisoning.^32^ *In situ* Raman spectroscopy is used to monitor material stability under inert, reducing, and oxidizing atmospheres at temperatures up to 1000 °C. Data show SVMO-19 to be stable under inert, reducing, and weakly oxidizing atmospheres. In air, however, SVMO-19 phase separates into a dominant scheelite phase (SrMoO_4_) and secondary Sr and V containing phases (SrVO_3_ and Sr_2_V_2_O_7_). TGA data corroborate these findings and set a 400 °C threshold for oxygen uptake. Isothermal Raman and TGA kinetic analyses of SVMO-19 degradation show clearly that scheelite formation is direct without intermediate steps and proceeds *via* 2 first order rate processes with the faster process describing surface decomposition and the latter reaction describing conversion of the bulk material.

## 2 Experimental Section

### 2.1 Sr_1.9_VMoO_6−δ_ (SVMO-19) Synthesis

Solid state synthesis techniques were developed to mimic previously reported procedures^29^ These solid-state methods combined SrCO_3_, VO_2_, and MoO_2_ powder precursors in distilled water. The solution was then mixed with an ultrasonic probe at a power output of 60%. To avoid separation of the precursors and water, liquid nitrogen was immediately introduced to flash freeze the aqueous mixture. The solidified mixture was lyophilized for 48 h with the results being a homogenized fine particulate powder. The powder was processed by hand using a mortar and pestle and placed in a Thermolyne 4800 box furnace to be calcined for 6 h at 1000 °C with a ramp to temperature of 5 °C/min and a return ramp to 25 °C of 10 °C/min. This calcination step removes carbonates and residual moisture from the freeze-dried powder and forms a mixture of SrMoO_4_ (Scheelite) and Sr_2_V_2_O_7_ phases. As the Mo^6+^ and V^5+^ oxidation states are immiscible and unable to form an oxidized solid-state perovskite, this calcination step serves as an intermediate solid cation mixing step toward perovskite formation *via* reduction.

Subsequent hand processing using a mortar and pestle was performed before forming 3/4″ diameter pellets through uniaxial pressing at 250 MPa. The pellets were reduced at 1100 °C in a forming gas atmosphere of 5%H_2_/95%Ar to produce SVMO and single perovskite secondary phases (SrVO_3_ and SrMoO_3_). Post reduction, the pellets were again processed by hand before being dispersed in ethanol and wet ball-milled for 48 h. The perovskite solvent mixture was air-dried and uniaxially pressed into 1/2″ pellets at 250 MPa and then sintered under N_2_ at 1200 °C to convert remaining perovskites to fully formed SVMO. In the final processing stage, the material (already at 1200 °C under N_2_) was then placed under a forming gas environment before having the temperature ramped to 1500 °C and then maintained at 1400 °C dwell for 12 h before cooling.

### 2.2 Chemical Stability Testing

The synthesized SVMO was tested to evaluate its phase and chemical stability under reducing atmospheres (5% H_2_ in N_2_), inert atmospheres, weakly oxidizing atmospheres (20% CO_2_) in Ar and humidified (3% H_2_O) argon, and an oxidizing atmosphere (air). All experiments were carried out at controlled-atmospheric conditions. Several analytical techniques were employed to characterize SVMO stability including XRD, Raman spectroscopy, and thermogravimetric analysis (TGA).

#### 2.2.1 X-ray Diffraction

X-ray powder diffraction (XRD) was utilized to confirm the double perovskite crystal structure in SVMO-19 samples. This was conducted using a Bruker D8 Advance powder X-ray diffractometer (PXRD) with Cu Kα radiation, operating at 40 kV and 40 mA, equipped with a Ni filter. The analysis utilized an angle step size of 0.02° over a scanning range of 5–80° to identify phases and assess phase purity. Rietveld refinement provided data about each sample’s phase purity.

#### 2.2.2 Raman Spectroscopy

*In situ* Raman spectroscopy, performed using continuous wave 488 nm excitation coupled into a Renishaw inVia spectrometer and a Linkam CCR1000 heating stage. Measurements were made as samples were heated from room temperature to 1000 °C in a controlled atmospheric environment. Raman spectra were collected at intervals of 100 °C, with a heating rate of 10 °C/min between temperatures and a dwell time of 15 min at temperature to equilibrate. Most spectra shown in this work resulted from 3 × 30-s accumulations. Incident laser powers were ∼30 mW with a ∼100 μm spot size using a 5× objective. Incident power at the sample surface was 0.1 mW.

#### 2.2.3 Thermogravimetric Analysis

Thermogravimetric analysis (TGA) was employed to characterize SVMO-19 thermal and chemical stability. Experiments were performed using a TA-TGA 5500 instrument, measuring weight loss/gain as a function of temperature in both inert (N_2_) and oxidizing (air) atmospheres. The analysis covered a temperature range from room temperature to 900 °C, with a heating rate of 5 °C/min and a flow rate of 25 mL/min. Measurements reproducibly measured mass changes as small as ±0.01%. Pseudoisothermal TGA measurements were performed by ramping the temperature as quickly as possible (50 °C/min) to the target temperature and then maintaining a constant temperature for the duration of the experiment.

## 3 Results and Discussion

### 3.1 Phase Composition and Crystal Structure of SVMO-19

Figure 1 shows the room-temperature X-ray diffraction pattern for the as-fabricated SVMO-19. The diffraction pattern exhibits sharp and well-defined peaks that are consistent with previous reports.^29^ These results confirm that the solid-state synthesis method described above can produce high purity, double perovskite materials.

**Figure 1:**
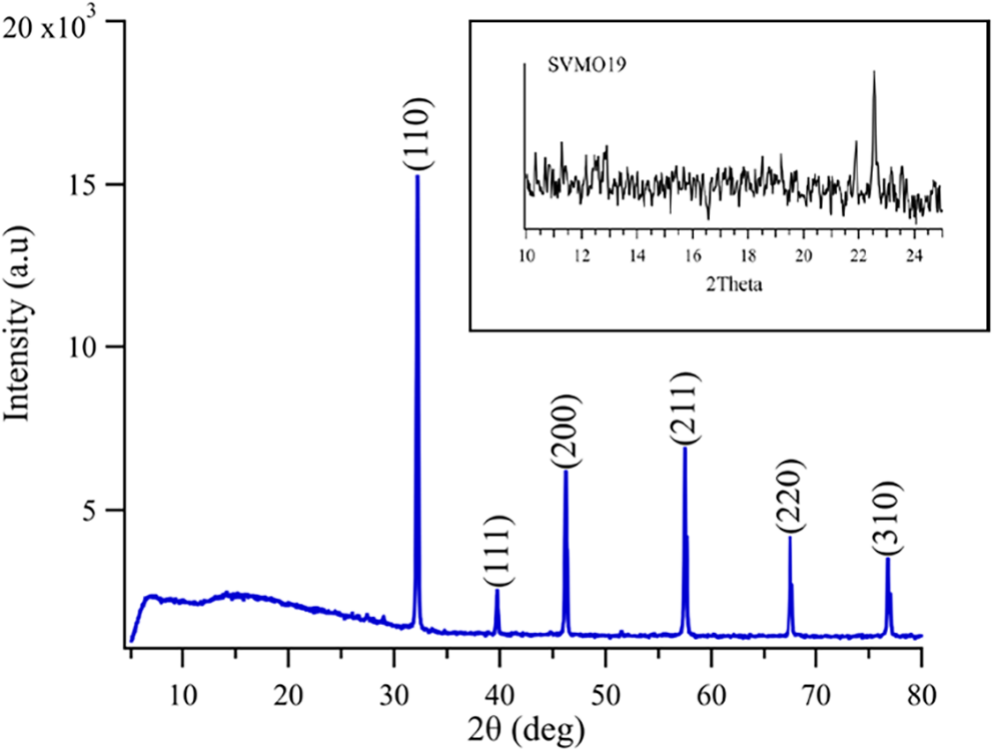
XRD patterns of SVMO-19 double-perovskite as-fabricated. Inset: XRD patterns of the SVMO-19 showing the expanded view of 2θ = 10 −24° with features assigned to secondary phases.

Rietveld refinement shows near-single phase purity with cubic SVMO content consistently ≥94%. (Residual materials include SrMoO_4_ (∼3%), lesser amounts of a tetragonal and hexagonal an isotropic lattice constant of 3.192 Å), again consistent with previous reports showing the presence of the *Pm*3̅*m* primitive perovskite structure. Examining the SVMO-19 diffraction patterns more closely, very weak features are observed at low 2θ (Figure 1, inset). These features, located approximately at 13°, 22°, 23°, have been cited as evidence of long-range single perovskite domain ordering.^29,33,34^ Features at these scattering angles could also arise from the small amount of secondary phases cataloged in the Rietveld refined structure.

### 3.2 SVMO-19 Stability in Inert, Reducing and Oxidizing Atmospheres

Secondary phases that form in double perovskites can compromise the material’s MIEC properties. Often, these secondary phases are crucial for understanding the material’s performance given that many are electronic and ionic insulating. To address SVMO’s susceptibility to secondary phase formation at the surface, we employed *in situ* Raman spectroscopy to monitor and characterize material stability under conditions common to SOFC and SOEC conditions.

Figure 2a shows Raman spectra of SVMO-19 over a temperature range from room temperature to 1000 °C in a 5% H_2_/N_2_ (forming gas) atmosphere. When exposed to forming gas, the Raman spectrum remains featureless, with the only noticeable change being a significant rise in the baseline at temperatures exceeding 800 °C. Similar behavior is observed when the material is subjected to argon and humidified argon (3% steam).

**Figure 2:**
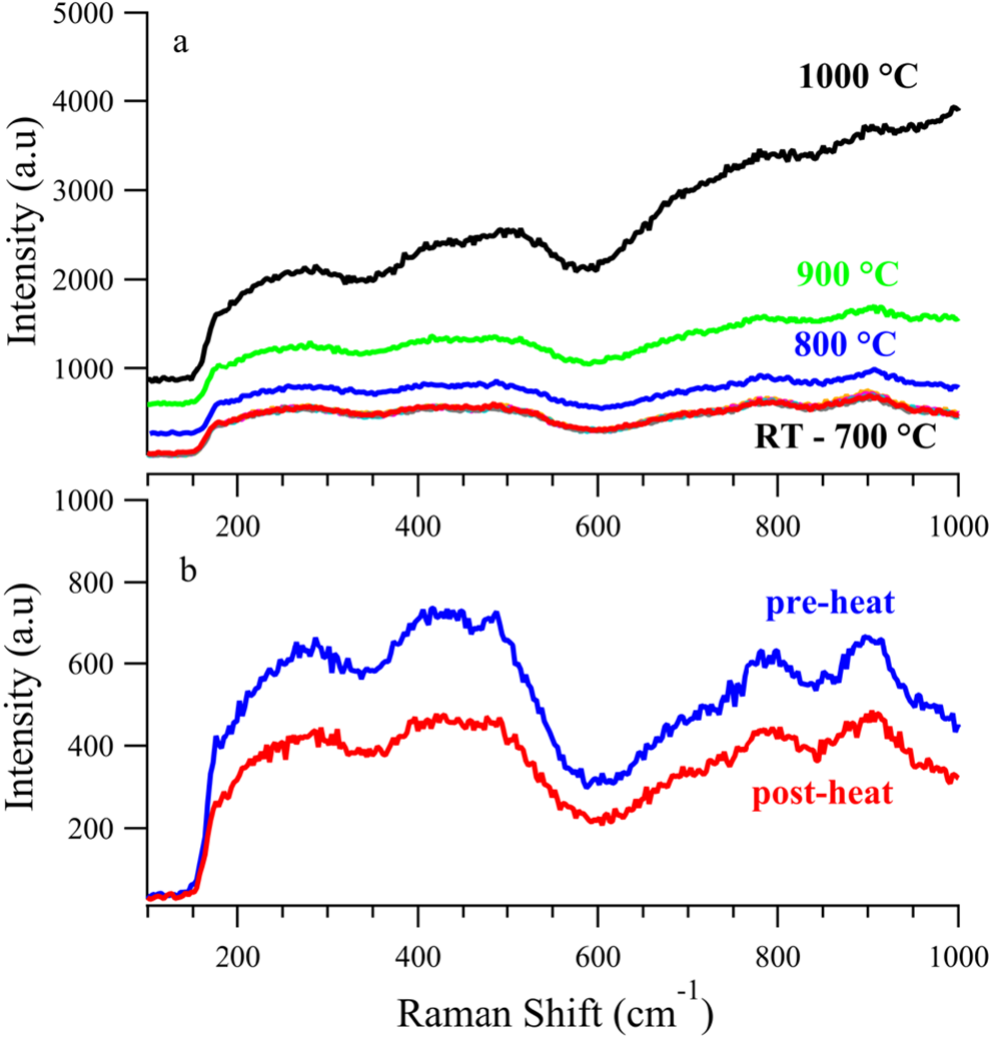
(a) Raman spectra of SVMO-19 in 5% H_2_ from room temperature to 1000 °C; (b) Raman spectra of SVMO-19 in 5% H_2_ pre- and post-heat.

The spectra in 2a are virtually identical except for a baseline that begins to rise at temperatures above 700 °C. However, even at 1000 °C, the spectrum remains largely featureless. Possible explanations for the rising baseline include background blackbody radiation from the heated sample and a change in the material’s reflectivity. These effects, however, are reversible as evidenced by the “before” and “after” spectra shown in Figure 2b. To within a small scaling factor, the pre- and post-heating spectra of SVMO-19 are almost identical, demonstrating that that SVMO-19 is thermally stable in reducing atmospheres. (Similar results were observed for experiments conducted in inert atmospheres (N_2_) as well as weakly oxidizing atmospheres (3% steam in N_2_)). Importantly, SVMO-19 in a 20% CO_2_ atmosphere (in N_2_) showed behavior equivalent to that under 5% H_2_. This result is significant because both CO_2_ and H_2_O are known to be weakly oxidizing at elevated temperatures, showing that SVMO-19 has some resistance to oxidation. (Results from the SVMO-19 thermal stability studies under N_2_ and N_2_ with 3% steam, and N_2_ are reported in Supporting Information). (Figure 3).

**Figure 3:**
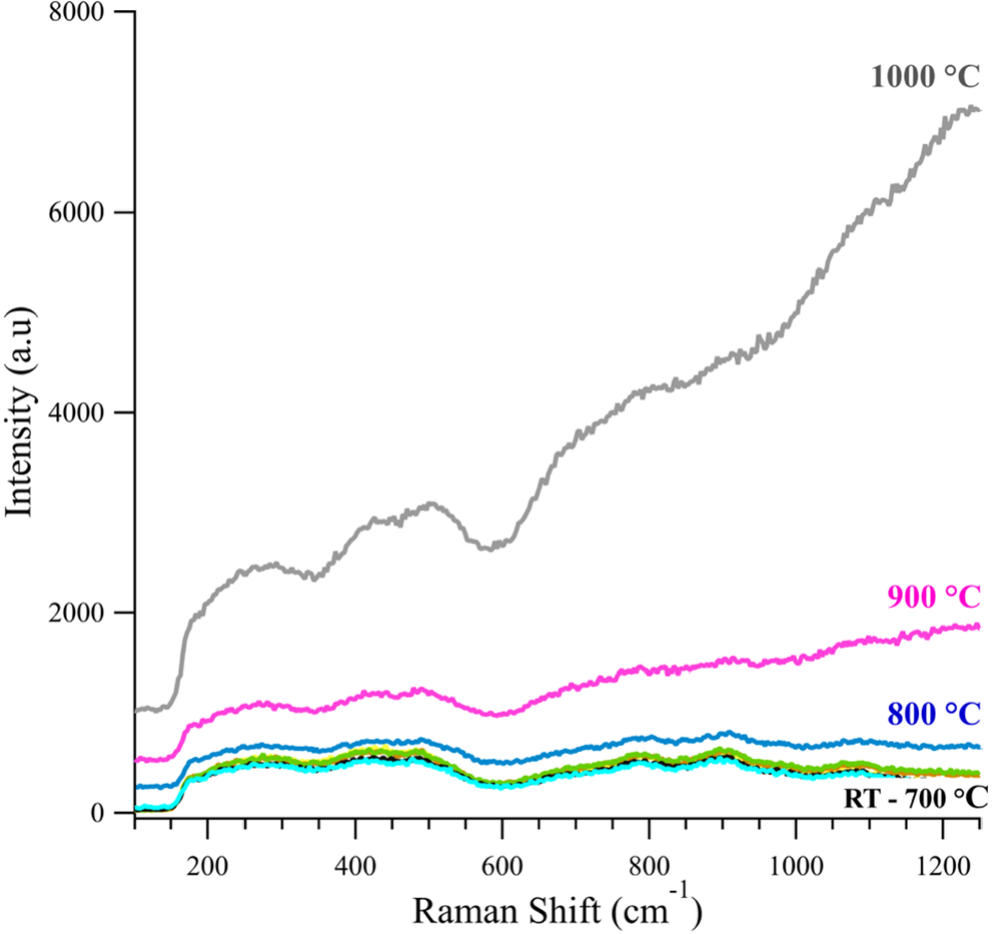
*In Situ* Raman spectra of SVMO-19 in 20% CO_2_ from room temperature to 1000 °C.

Unlike behavior observed in reducing, inert, and weakly oxidizing atmospheres, SVMO-19 is distinctly unstable in air at elevated temperatures. Figure 4a shows the evolution of SVMO-19 as the sample temperature is raised in 100° increments to 1000 °C. At temperatures above 600 °C in air, a prominent Raman peak is observed near 876 cm^–1^. Additionally, the Raman spectrum of SVMO19 shows several other peaks appearing at approximately 825, 776, 358, and 322 cm^–1^. Based on findings reported by Son et al.,^31^ these features are assigned to the scheelite phase, SrMoO_4_. A consequence of SVMO’s decomposition in air at elevated temperatures implies that this material will not be effective as the oxygen electrode in SOFCs and SOECs.

**Figure 4:**
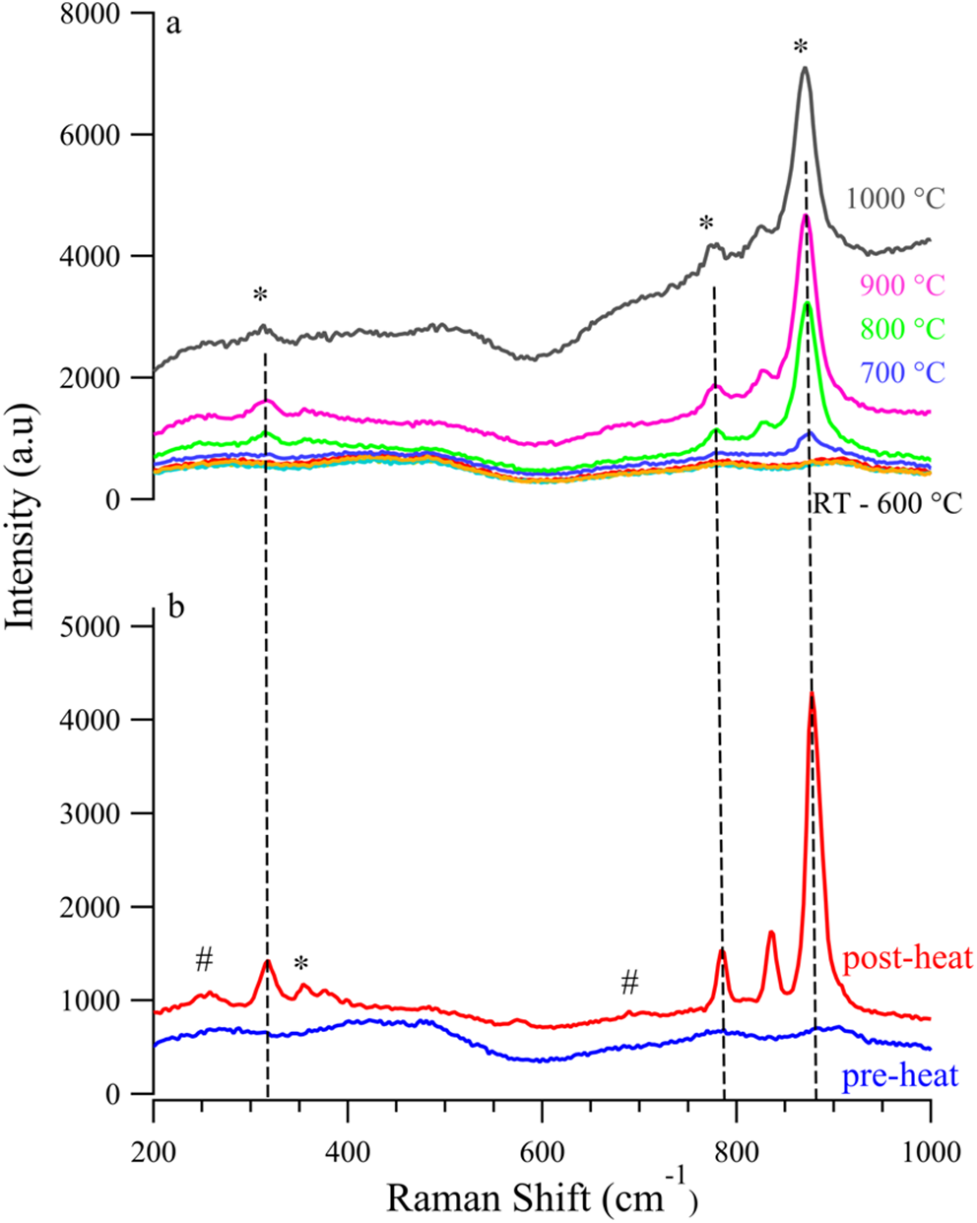
(a) *In situ* Raman spectra of SVMO-19 in air from room temperature to 1000 °C; (b) Raman spectra of SVMO-19 in air pre- and postheat (* = SrMoO_4_; # = SrVO_3_).

Earlier studies by Porto and Scott^35^ provided a detailed analysis of the Raman modes associated with the scheelite-type structure of SrMoO_4_ and their results indicated that the most intense Raman peak, occurring at 888 cm^–1^, corresponds to the totally symmetric stretching motion of the tetrahedral MoO_4_ group. In Figure 4a the observed peak at 876 cm^–1^ is assigned to SrMoO_4_, and its growth signifies secondary phase formation in SVMO-19. A Raman spectrum of this sample after cooling back to room temperature shows the SrMoO_4_ vibrational features clearly. Additional small features at 685 cm^–1^ and 259 cm^–1^ are assigned tentatively to the bending vibrations of VO_3_ groups associated with the single perovskite SrVO_3_.^36^

To assess how fast SrMoO_4_ forms, we performed an isothermal Raman experiment. In this experiment, SVMO-19 was heated to 700 °C under forming gas. After reaching the target temperature and dwelling for 15 min with no change in the spectrum, the atmosphere was switched from forming gas to air (assigned as *t*_0_ = 0), and spectra were acquired every 5 min. (Residence times in the gas manifold are ≤20 s). Results from this experiment are shown in Figure 5. Figure 5a shows selected spectra acquired at different times after *t*_0_. Figure 5b plots the intensity of the primary SrMoO_4_ feature at 876 cm^–1^ as a function of time.

**Figure 5:**
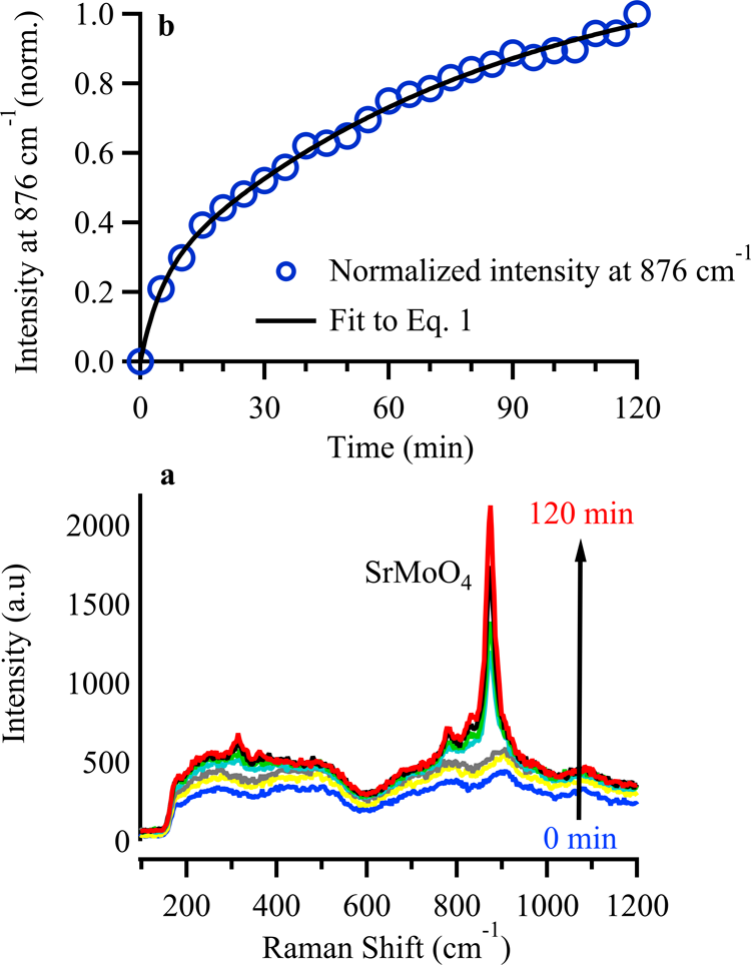
(a) *In Situ* Raman spectra of SVMO-19 in 5% H_2_ and air from room temperature to 700 °C; (b) kinetic trace showing the normalized intensity of the SrMoO_4_ secondary phase after exposure to air at 700 °C.

Raman spectroscopy reveals that SVMO-19 under air at 700 °C takes up O_2_ to form SrMoO_4_. The emergence and growth of SrMoO_4_ provide direct evidence that SVMO-19 is unstable under conditions commonly encountered as the oxygen electrode in solid oxide electrochemical cells. The data in Figure 5b can fit to a sum of two single exponential functions having characteristic rate constants, *k*_1_ and *k*_2_

1 $$\mathrm{Int}\left(t\right)=A_{0}+A_{1}e^{-k_{1}t}+A_{2}e^{-k_{2}t}$$

Equation 1 suggests that SrMoO_4_ forms *via* two first-order (or pseudo first order) processes with one rate measurably faster than the other. This hypothesis will be examined in Section 4 below.

#### 3.2.1 Thermal Analysis

Thermal gravimetric analysis (TGA) was used to independently test the thermal stability of SVMO-19 in inert and air atmospheres. Figure 6 shows TGA traces of SVMO-19 in both N_2_ and air as the sample temperature was raised to 900 °C at 5 °C/min.

**Figure 6:**
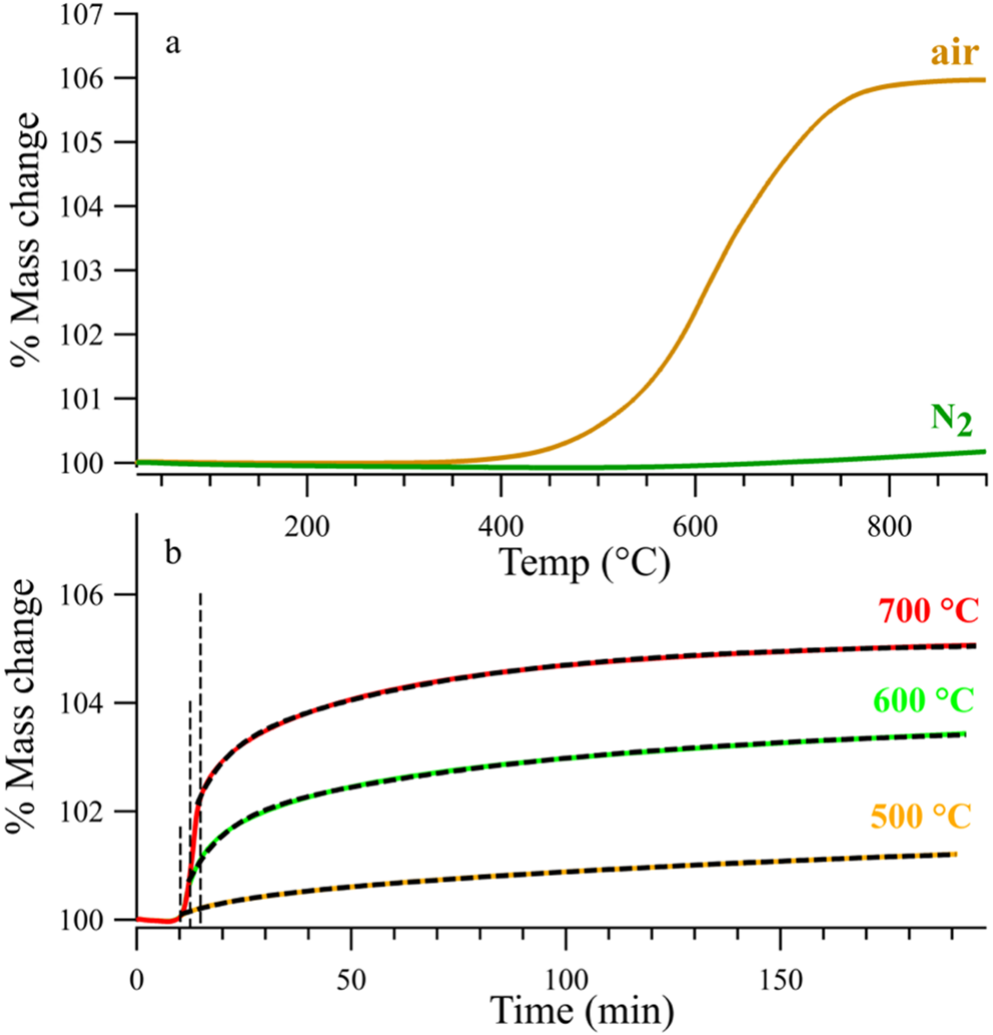
(a) TGA curves of SVMO-19 in N_2_ and air from 25 to 900 °C (b) Isothermal TGA curves of SVMO-19 in air at 500 °C, 600 and 700 °C. The dashed vertical lines correspond to the time when the sample has reached its target temperature. The dashed lines superimposed on the data correspond to fits generated by eq 1.

Figure 6a shows that SVMO-19 is stable under N_2_ at temperatures up to 900 °C. The slight gain in mass (0.1%) is associated with instrument drift and post-mortem Raman analysis of the sample showed that it remained SVMO-19. In contrast, SVMO-19 being heated in air shows mass accumulation beginning at ∼400 °C with sample mass growing steadily before asymptotically leveling off near 750 °C.

To test if the rate of mass gain observed in the TGA experiment mirrored that in the isothermal Raman experiment (Figure 5b), we performed a “pseudo”-isothermal experiment using TGA. In this experiment, the TGA was ramped as quickly as possible (50 °C/min) to 700 °C in air and then the temperature was held constant. Results from this experiment are shown in Figure 6b. The 700 °C trace shows that the sample begins acquiring mass before reaching its target temperature, but the last ∼40% of the mass gain happens isothermally. Superimposed on the isothermal TGA data is a fit to the same double exponential function shown in eq 1. Again, the data fit exceptionally well to this functional form, implying that the mass gain corresponds directly to SrMoO_4_ formation. This same method was repeated for a *T*_max_ of 600 and 500 °C and the data are included in Figure 6b. Again, the lower temperature data also follow the sum of two exponential decays.

## 4 Discussion

Several questions emerge from the data presented above:

- How does oxygen uptake lead to SVMO-19 degradation?
- Do the kinetic rates observed in the isothermal Raman and TGA experiments correspond to the same processes?
- In both experiments, what physical phenomena do the two kinetic processes describe?

The first of these questions is easiest to answer. Raman data show that a major product formed from SVMO-19 degradation is SrMoO_4_. Raman data also show SrVO_3_ in the sample postheating. (SrVO_3_ has a very small Raman scattering cross section. Even considerable quantities of this material would only provide a weak Raman signature). Finally, XRD patterns from the sample after it has been heated in air show an abundant pyrochlore phase, Sr_2_V_2_O_7_. (Figure 7)

**Figure 7:**
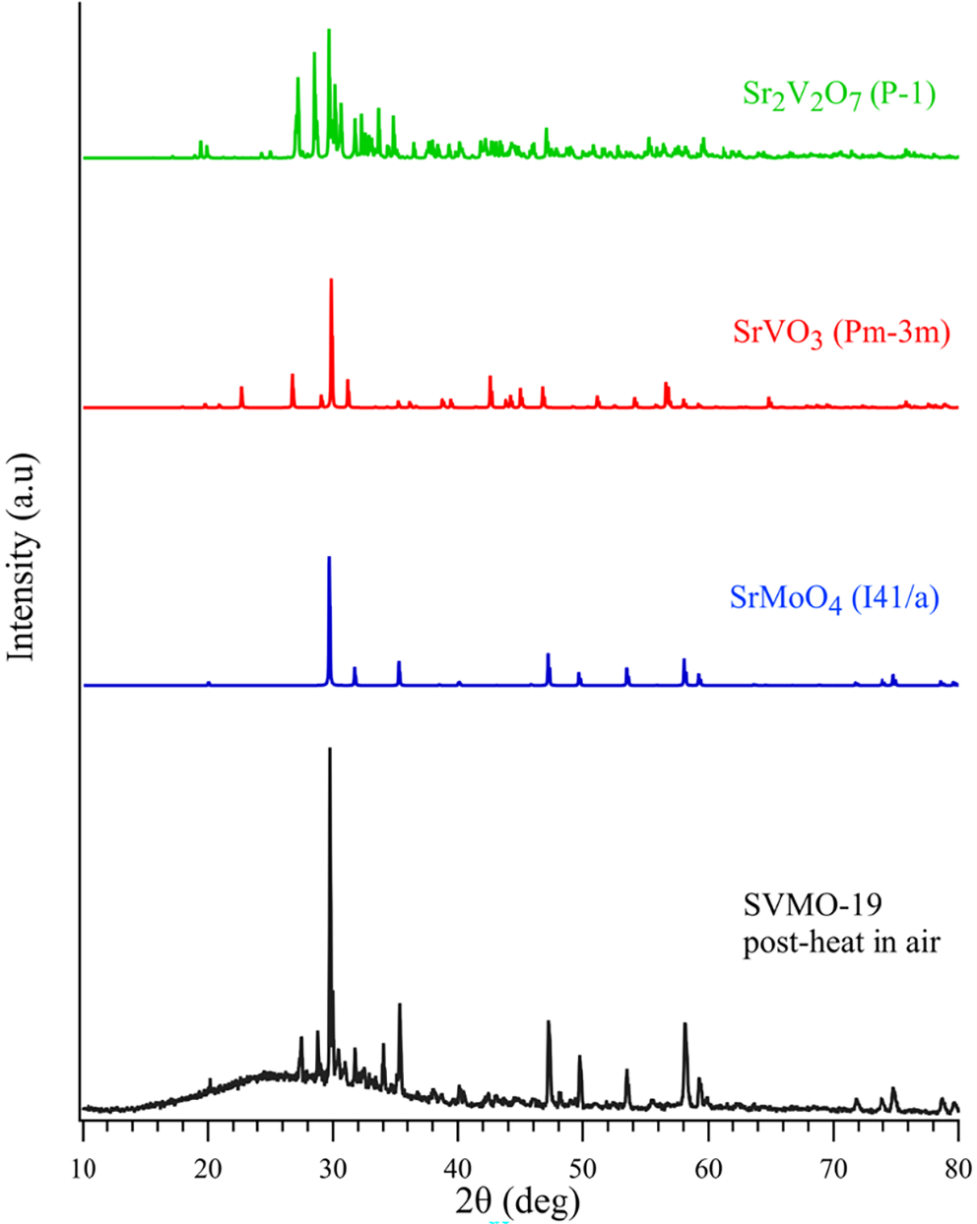
XRD pattern from SVMO-19 sample after being heated to 800̊C in air. Rietveld refinement identifies 3 distinct secondary phases.

The XRD data in Figure 7 show definitively that SVMO-19 converts completely to SrMoO_4_, SrVO_3_, and Sr_2_V_2_O_7_ with no measurable SVMO remaining in the sample.

Simple stoichiometric considerations require

2 $$3Sr_{2.0}VMoO_{6-\delta }+2O_{2}\to 3SrMoO_{4}+SrVO_{3}+Sr_{2}V_{2}O_{7}$$

For A-site deficient SVMO-19, we propose that the perovskite is oxygen deficient (Sr_1–*x*_VO_3−δ_) given that a perfect perovskite structure does not have any Raman active modes and instead requires defects or grain boundaries to induce a nonzero polarizability. These results are consistent with those reported by Son et al.^31^ who reported scheelite, and perovskite phases formed during Sr_2_FeMoO_6−δ_ oxidation in air.

The second question—do the isothermal Raman measurements tracking SrMoO_4_ growth and the TGA measurements recording mass uptake report the same processes?—is an interesting one. TGA data contain no material specificity. Raman data, on the other hand, measure the real-time growth of SrMO_4_. After reconciling units, we calculated rate constants (*k*_i_) for the isothermal Raman intensity plot at 700 °C shown in Figure 5b and the double exponential rate constants for the three isothermal TGA plots shown in Figure 6b. The results are reported in Table 1.

**Table 1:** Rate Constants Determined from Fitting Data in Figures 6b and 6b to Equation 1 ^a^

method	temp °(C)	*k*_1_ (s^–1^)	*k*_2_ (s^–1^)	*A*_0_	*A*_1_	*A*_2_
Raman	700	3.4 × 10^–3^	2.1 × 10^–4^	1.18	–0.96	–0.22
TGA	700	2.3 × 10^–3^	3.4 × 10^–4^	1.01	–0.53	–1.73
TGA	600	2.1 × 10^–3^	2.0 × 10^–4^	1.05	–0.61	–1.43
TGA	500	1.3 × 10^–3^	9.5 × 10^–5^	1.38	–1.14	–0.45

a Typical uncertainties in *k*_1_ are ± 20%; typical uncertainties in *k*_2_ are ± 5%.

This analysis assumed that the rates were described by pseudo-first order rate constants where the O_2_ partial pressure is taken as a constant. Given similarities in rate constants for the 700 °C Raman and TGA data, we propose that the observables measured in each experiment describe the same physical processes. This conclusion is important because it implies oxygen uptake is rate limiting when SVMO-19 oxidizes in air and that SrMoO_4_ forms at the same rate that O_2_ is being absorbed.

To address the third question posed above—to what physical processes do these rates correspond?—we first considered other possible models that might be applied to analyze phase transformations in solid state samples. The Avrami model, widely used for phase transformations in materials systems, predicts sigmoidal kinetics and is represented as follows

3 $$f\left(t\right)=1-\exp\left(-kt^{n}\right)$$

where *f*(*t*) is the fraction transformed at time *t*, and *k* and *n* are kinetic parameters.^37^ The Avrami exponent *n* provides mechanistic insight: values near 1 suggest surface-controlled reactions or instantaneous nucleation with one-dimensional growth, while 0 < *n* < 1 indicates diffusion-limited or constrained kinetics. In this regime, the rate is limited by the slow movement of O^2–^ions through the lattice rather than by surface reaction or nucleation. The rate constant *k* reflects the combined effects of nucleation and growth processes.^37^

Empirically, we found that the double exponential function (eq 1) representing two independent first order kinetic processes fit the data better than the Avrami function (eq 3), especially at the lowest temperature (500̊C). We rationalize this observation in terms of the role played by oxide vacancies in facilitating O^2–^ diffusion in A-site deficient materials. Additionally, we note that in order for SVMO-19 to oxidize, excess oxygen must be present, meaning that the interior of the samples used in this work cannot oxidize to form secondary phases until O^2–^ ions diffuse into the material’s bulk.

Removing Sr^2+^ ions from the A-site produces Sr vacancies (V_Sr_^″^) and charge–compensating O^2–^ vacancies (V_O_^••^) according to

4 $${\mathrm{Sr}}_{\mathrm{Sr}}^{\times }+O_{O}^{\times }\to V_{\mathrm{Sr}}^{\prime \prime }+V_{O}^{••}+{\mathrm{SrO}}_{\left(s\right)}$$

These vacancies provide pathways that promote O^2–^ diffusion. Under oxidizing conditions, molecular oxygen reacts rapidly at the surface of to fill these pre-existing vacancies

5 $$\frac{1}{2}O_{2\left(g\right)}+V_{O}^{••}+{2e}^{\prime }\to O_{O}^{\times }$$

Because these defect sites are already present, we expect that SVMO-19 oxidation forms SrMoO_4_ and other secondary phases rapidly. In the double exponential model, secondary phases will form in the bulk after the oxides diffuse into the bulk with a similar activation energy. In the Avrami model, oxide diffusion then becomes rate limiting and the material transformation (from SVMO-19 to SrMoO_4_ and Sr_2_V_2_O_7_) in the bulk happens as soon as oxides arrive.

When the Avrami constant, *n*, approaches unity as is the case when fitting the isothermal TGA data at 500 °C to an Avrami model, oxidation is assumed to happen spontaneously at multiple sites throughout the material rather than with a single diffusion front.^38–40^ Such a situation requires that the oxide responsible for driving the phase transition *already be present* in the material. This situation does not describe conditions relevant for SVMO-19, where the oxides must first diffuse into the interior before the SVMO-19 oxidation can occur.

Given the similarity in activation energies determined from the double exponential fit and the inability of an Avrami model to fit the low temperature data, we choose to use the former model to describe the isothermal TGA and Raman data. The two first order processes are tentatively assigned to transformation of the surface (and near-surface) of SVMO-19 into its oxidized secondary phases and followed by the slower transformation of the bulk material after the diffusing oxides have arrived. This model implicitly assumes that oxidation in the bulk material is slower than oxidation of the surface. We acknowledge that this description is speculative, but it suggests several testable hypotheses that we will explore in subsequent work that examines SVMO-19 stability as a function of A-site deficiency (and B-site composition).

The double exponential kinetic model provides significantly better fits (Figure 8a) across all temperatures, accurately capturing both the initial and long-term kinetics of the transformation. In contrast, superimposed on the isothermal TGA data at 500, 600, and 700 °C (Figure 8b) are fits to the Avrami kinetic model (eq 3). We note that to fit the isothermal TGA data to an Avrami model, data needed to be scaled and normalized to 1.0 before eq 3 could be applied. While the Avrami model captures the general sigmoidal behavior of the mass uptake, the agreement with the experimental data is limited. Discrepancies between the two different models suggest that mass uptake and phase transformation is better described by two distinct rate-limiting steps rather than a single, unified transformation mechanism assumed in the Avrami approach.

**Figure 8:**
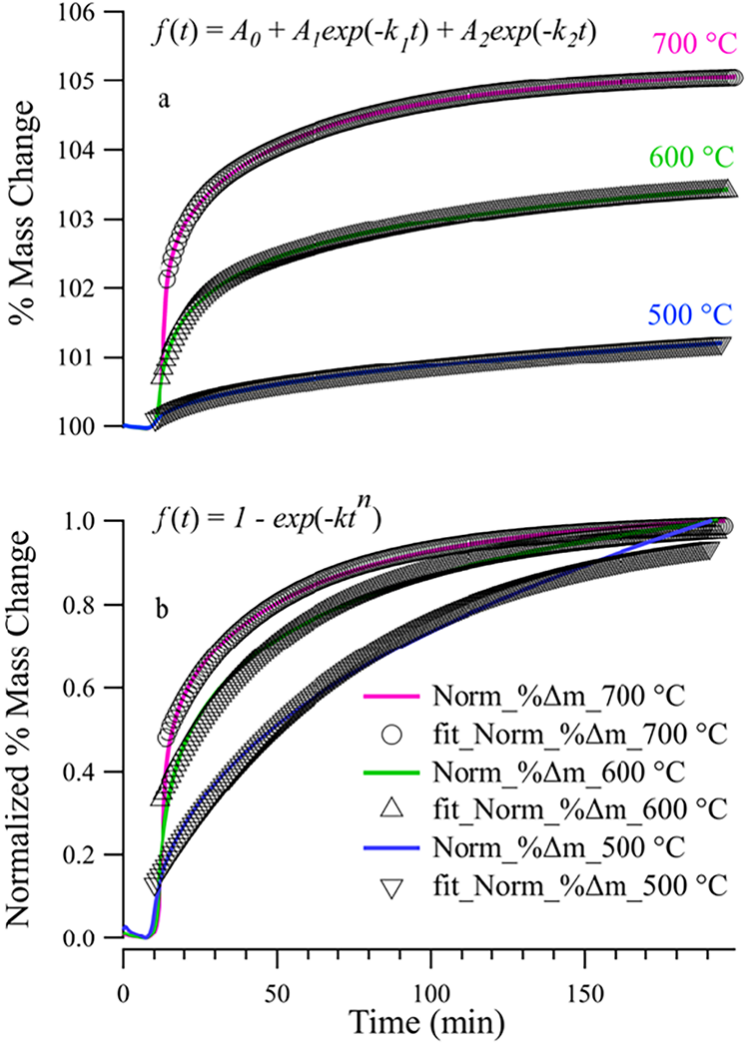
Isothermal TGA curves of SVMO-19 in air at 500 °C, 600 and 700 °C with fits to (a) the double exponential kinetic, and (b) the Avrami kinetic models.

Following unit reconciliation, the Avrami rate constants (*k*) and exponents (*n*) were determined for the isothermal Raman intensity data at 700 °C (Figure 5b) and for the isothermal TGA data at 500 °C, 600 °C, and 700 °C (Figure 8b). Calculated kinetic parameters are reported in Table 2.

**Table 2:** Rate Constant and Exponent Determined from Fitting Data in Figure 8b to Equation 3 ^a^

method	temp °(C)	*k* (s^–1^)	*n*
Raman	700	4.5 × 10^–2^	0.84
TGA	700	9.9 × 10^–2^	0.72
TGA	600	5.6 × 10^–2^	0.79
TGA	500	1.4 × 10^–2^	1.01

a Typical uncertainties in rate constants are less than ± 25%.

Several observations from Table 2 stand out. First, the Avrami rate constants are an order of magnitude larger than the rate constants determined from the double exponential fit. Importantly, the difference between the Raman and TGA rate constants at 700 °C is more than a factor of 2, compared to the ∼50% differences found from the double exponential model. Second, the Avrami exponent, n, is less than unity for kinetic data at 600 and 700 °C and equal to unity for the isothermal TGA data taken at 500 °C. According to the conventional interpretation of the Avrami exponent, values less than unity correspond to diffusion-limited processes, while the 1.0 value at 500 °C would indicate a change in SVMO-19 decomposition to a surface-controlled mechanism.

With temperature dependent rate constants for both models—double exponential and Avrami—we performed Arrhenius analyses for both data sets. For the double exponential model, plotting ln­(*k*_*i*_) *versus* 1/*T*_*i*_ for *k*_1_ and *k*_2_ yielded two linear trends with similar slopes corresponding to activation energies *E*_a,1_ = 0.65 ± 0.19 eV and *E*_a,2_ = 0.48 ± 0.01 eV, respectively (Figure 9).

**Figure 9:**
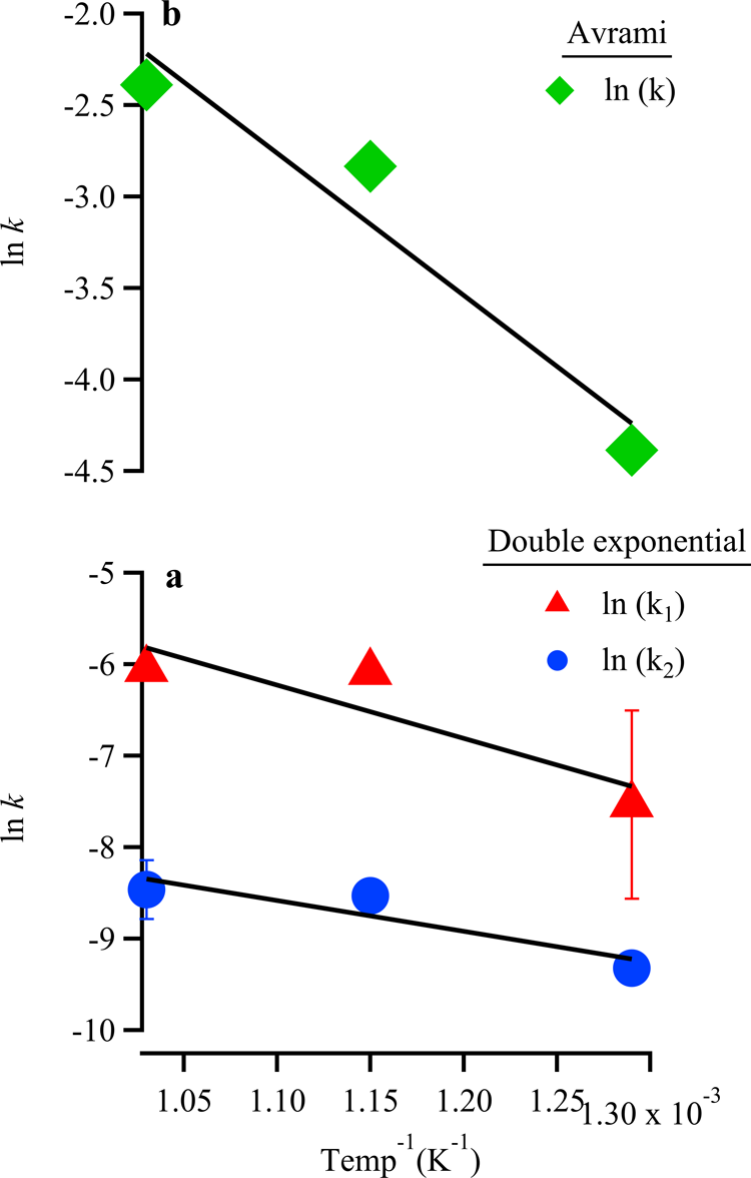
Arrhenius plots for SVMO-19 kinetic data from isothermal TGA measurements shown in Figure 6. Data in panel (A) correspond to a double exponential function describing two independent first order kinetic processes. (See eq 1). Data in panel (B) correspond to rate constants derived from an Avrami model. (See eq 3). Experiments at each temperature were carried out a minimum of 4 times and the uncertainties represent two standard deviations. Note the break in scale between panels (A, B).

A similar analysis of the Avrami rate constants produced a single linear fit, yielding an activation energy of *E*_a_ = 0.62 ± 0.12 eV (Figure 9). We note that the activation energies calculated from all three sets of rate constants (double exponential fast, double exponential slow, Avrami) are quite similar. This observation shows that the energetics of SVMO-19 decomposition are largely insensitive to the model used to describe the process. Based on the goodness of fits shown in Figure 8, we propose that the double exponential model is more appropriate to describe the processes. Furthermore, similarities between *E*_a,1_ and *E*_a,2_ to within experimental uncertainty suggest that the two kinetic processes are, themselves, also similar. Our hypothesis is that the faster rate constant, *k*_1_, corresponds to SVMO-19 decomposition and secondary phase formation at the material surface where reaction with ambient O_2_ happens quickly, albeit with a higher activation energy. In this scenario, *k*_2_ corresponds to SVMO-19 decomposition and secondary phase formation in the bulk. This second process should be slower given that it requires atomic restructuring that is more restricted than on the sample surface. Additionally, the pre-exponential factors *A*_1_ and *A*_2_ provide insight into the respective temperature ranges over which SVMO decomposition takes place. At elevated temperatures, the coefficient corresponding to the faster rate (*A*_1_) is less than the slower rate coefficient (*A*_2_). These numbers are consistent with more decomposition taking place in the bulk although the rate of reaction is slower. At 500 °C, the relative magnitudes reverse with *A*_1_ being greater than *A*_2_. We propose that at lower temperatures, mass transport into the bulk is simply too slow and measurements are sensitive primarily to surface transformations. In the case of the isothermal Raman measurements at 700 °C, *A*_1_ is again greater than *A*_2_, but Raman measurements are necessarily more sensitive to changes in a material’s surface and near surface composition than to changes occurring in the bulk.

## 5 Conclusions

Double perovskite materials are attractive candidates to serve as electrodes in high temperature solid oxide electrochemical devices. Before they can be integrated into this technology, however, individual materials must be evaluated in terms of their electrocatalytic potential, their resilience to contamination, and their stability to changes in ambient atmosphere. These assessments need to happen at relevant operating temperatures and in atmospheres representative of those the materials will encounter under operational conditions.

SVMO holds promise given earlier reports of high conductivities.^29^ In the current work, we used a combination of independent methods to assess SVMO-19’s stability under a variety of thermal and atmospheric conditions. In doing so, we have identified conditions that support SVMO-19’s sustained stability and conditions that lead to irreversible degradation. *In situ* Raman measurements demonstrate that SVMO-19 is stable up to 1000 °C under reducing atmospheres as well as weakly oxidizing atmospheres containing CO_2_ and H_2_O. This latter condition implies that SVMO-19 should be suitable for CO_2_ electrolysis and CO_2_/H_2_O coelectrolysis. SVMO, even as a fuel electrode could be resilient to fuel flow disruptions. Under strongly oxidizing atmospheres, SVMO rapidly separates into secondary phases including SrMoO_4_, SrVO_3_, and Sr_2_V_2_O_7_. A detailed examination of this decomposition process shows that it most closely follows a kinetic mechanism containing two independent first order (or pseudo first order) processes. The activation energies for these two processes are 0.53 and 0.70 eV. The similarity between these two values implies that the two processes describe similar transformations, ones that we tentatively assign to surface and bulk material reactions. More importantly, these findings demonstrated clearly that while SVMO-19 may be suitable for use as a fuel electrode in solid oxide cells (including under weakly oxidizing conditions), its utility as an oxygen electrode is limited given the material’s propensity to form secondary phases at high temperature in air.

## Supplementary Material



## Notes

*In situ* Raman spectra up to 1000 °C of SVMO-19 in N_2_, N_2_ with 3% H_2_O, and N_2_ with 20% CO_2_; room temperature XRD patterns of SVMO-19 as fabricated, after heating to 1000 °C under forming gas, and after heating to 1000 °C under air; kinetic traces of SVMO-19 based on Raman data acquired at 700 °C under cycling redox conditions (forming gas–air–forming gas–air) (PDF)

## References

[1] Vafaeenezhad S., Hanifi A. R., Laguna-Bercero M. A., Etsell T. H., Sarkar P. (2022). Microstructure
and long-term stability of Ni–YSZ anode supported fuel cells:
a review. Mater. Futures.

[2] Zhang J., Barreau M., Dintzer T., Haevecker M., Teschner D., Efimenko A., Luo W., Zafeiratos S. (2024). Unveiling
Key Interface Characteristics of Ni/Yttria-Stabilized Zirconia Solid
Oxide Cell Electrodes in H2O Electroreduction Using Operando X-ray
Photoelectron Spectroscopy. ACS Appl. Mater.
Interfaces.

[3] Kullmann F., Schwiers A., Juckel M., Menzler N., Weber A. (2024). Enhancement
of Performance and Sulfur Tolerance of Ceria-Based Fuel Electrodes
in Low Temperature SOFC. J. Electrochem. Soc..

[4] Calkins W. H. (1987). Investigation
of organic sulfur-containing structures in coal by flash pyrolysis
experiments. Energy Fuels.

[5] Cheng Z., Wang J.-H., Choi Y., Yang L., Lin M.-C., Liu M. (2011). From Ni-YSZ to sulfur-tolerant
anode materials for SOFCs: electrochemical
behavior, in situ characterization, modeling, and future perspectives. Energy Environ. Sci..

[6] Gong M., Liu X., Trembly J., Johnson C. (2007). Sulfur-tolerant anode materials for
solid oxide fuel cell application. J. Power
Sources.

[7] Hua B., Li M., Chi B., Jian L. (2014). Enhanced electrochemical performance
and carbon deposition resistance of Ni–YSZ anode of solid oxide
fuel cells by in situ formed Ni–MnO layer for CH 4 on-cell
reforming. J. Mater. Chem. A.

[8] Koh J.-H., Yoo Y.-S., Park J.-W., Lim H. C. (2002). Carbon deposition
and cell performance of Ni-YSZ anode support SOFC with methane fuel. Solid State Ionics.

[9] Niakolas D. K. (2014). Sulfur
poisoning of Ni-based anodes for Solid Oxide Fuel Cells in H/C-based
fuels. Appl. Catal., A.

[10] Zhan Z., Barnett S. A. (2005). An octane-fueled
solid oxide fuel cell. Science.

[11] Song Y., Zhou Z., Zhang X., Zhou Y., Gong H., Lv H., Liu Q., Wang G., Bao X. (2018). Pure CO 2 electrolysis
over an Ni/YSZ cathode in a solid oxide electrolysis cell. J. Mater. Chem. A.

[12] Ebbesen S. D., Graves C., Hauch A., Jensen S. H., Mogensen M. (2010). Poisoning
of solid oxide electrolysis cells by impurities. J. Electrochem. Soc..

[13] Lv H., Lin L., Zhang X., Gao D., Song Y., Zhou Y., Liu Q., Wang G., Bao X. (2019). In situ exsolved FeNi 3 nanoparticles
on nickel doped Sr 2 Fe 1.5 Mo 0.5 O 6– δ perovskite
for efficient electrochemical CO 2 reduction reaction. J. Mater. Chem. A.

[14] Fagg D., Kharton V., Frade J., Ferreira A. (2003). Stability and mixed
ionic–electronic conductivity of (Sr, La)­(Ti, Fe) O3–
δ perovskites. Solid State Ionics.

[15] Chen X., Liu Q., Chan S., Brandon N., Khor K. A. (2007). High-performance
cathode-supported SOFC with perovskite anode operating in weakly humidified
hydrogen and methane. Fuel Cells Bull..

[16] Tao S., Irvine J. T., Kilner J. A. (2005). An efficient solid oxide fuel cell
based upon single-phase perovskites. Adv. Mater..

[17] Mukundan R., Brosha E. L., Garzon F. H. (2004). Sulfur tolerant anodes for SOFCs. Electrochem. Solid-State Lett..

[18] Fu X.-Z., Melnik J., Low Q.-X., Luo J.-L., Chuang K. T., Sanger A. R., Yang Q.-M. (2010). Surface
modified Ni foam as current
collector for syngas solid oxide fuel cells with perovskite anode
catalyst. Int. J. Hydrogen Energy.

[19] Huang Y.-H., Dass R. I., Denyszyn J. C., Goodenough J. B. (2006). Synthesis
and characterization of Sr2MgMoO6– δ: an anode material
for the solid oxide fuel cell. J. Electrochem.
Soc..

[20] Huang Y.-H., Dass R. I., Xing Z.-L., Goodenough J. B. (2006). Double
perovskites as anode materials for solid-oxide fuel cells. Science.

[21] Marina O. A., Canfield N. L., Stevenson J. W. (2002). Thermal,
electrical, and electrocatalytical
properties of lanthanum-doped strontium titanate. Solid State Ionics.

[22] Qiu P., Yang X., Wang W., Wei T., Lu Y., Lin J., Yuan Z., Jia L., Li J., Chen F. (2020). Redox-reversible
electrode material for direct hydrocarbon solid oxide fuel cells. ACS Appl. Mater. Interfaces.

[23] Song Y., Zhong Q., Tan W., Pan C. (2014). Effect of cobalt-substitution
Sr2Fe1. 5-xCoxMo0. 5O6-δ for intermediate temperature symmetrical
solid oxide fuel cells fed with H2-H2S. Electrochim.
Acta.

[24] Xiao G. L., Liu Q., Zhao F., Zhang L., Xia C., Chen F. (2011). Sr2Fe1.5Mo0.5O6
as cathodes for intermediate-temperature solid oxide fuel cells with
La0.8Sr0.2Ga0.87Mg0.13O3 electrolyte. J. Electrochem.
Soc..

[25] Ding H., Tao Z., Liu S., Yang Y. (2016). A redox-stable direct-methane solid
oxide fuel cell (SOFC) with Sr2FeNb0. 2Mo0. 8O6– δ double
perovskite as anode material. J. Power Sources.

[26] Tao S., Irvine J. T. (2003). A redox-stable efficient
anode for solid-oxide fuel
cells. Nat. Mater..

[27] Zhang M., Li Y., Du Z., Zhang Y., Zhao H. (2024). Double perovskite Sr2FeMo0.
6Mg0. 25Ga0. 15O6– δ as high-performance fuel electrode
for reversible solid oxide cell. J. Power Sources.

[28] Gou M., Ren R., Sun W., Xu C., Meng X., Wang Z., Qiao J., Sun K. (2019). Nb-doped Sr2Fe1.
5Mo0. 5O6-δ
electrode with enhanced stability and electrochemical performance
for symmetrical solid oxide fuel cells. Ceram.
Int..

[29] Childs N. B., Weisenstein A., Smith R., Sofie S., Key C. (2013). Electrical
conductivity of Sr2– xVMoO6– y (x= 0.0, 0.1, 0.2) double
perovskites. J. Appl. Phys..

[30] Huang Y.-H., Liang G., Croft M., Lehtimaki M., Karppinen M., Goodenough J. B. (2009). Double-perovskite anode materials
Sr2MMoO6 (M= Co, Ni) for solid oxide fuel cells. Chem. Mater..

[31] Son L. H., Phuc N. X., Phuc P. V., Hong N. M., Hong L. V. (2001). Observation
of phase decomposition of Sr2FeMoO6 by Raman spectroscopy. J. Raman Spectrosc..

[32] Niu B., Jin F., Fu R., Feng T., Shen Y., Liu J., He T. (2018). Pd-impregnated
Sr1. 9VMoO6−δ double perovskite as an
efficient and stable anode for solid-oxide fuel cells operating on
sulfur-containing syngas. Electrochim. Acta.

[33] Shimada T., Nakamura J., Motohashi T., Yamauchi H., Karppinen M. (2003). Kinetics and
thermodynamics of the degree of order of the B cations in double-perovskite
Sr2FeMoO6. Chem. Mater..

[34] Woodward P. M. (1997). Octahedral
tilting in perovskites. I. Geometrical considerations. Acta Crystallogr., Sect. B:Struct. Sci..

[35] Porto S. P. S., Scott J. (1967). Raman Spectra of CaW
O 4, SrW O 4, CaMo O 4, and SrMo
O 4. Phys. Rev..

[36] Botto I. L., Baran E. J., Pedregosa J. C., Aymonino P. J. (1979). The vibrational
spectrum of barium divanadate. Monatsh. Chem.-Chem.
Mont..

[37] Fanfoni M., Tomellini M. (1998). The johnson-mehl-avrami-kohnogorov
model: a brief review. Il Nuovo Cimento D.

[38] Avrami M. (1941). Granulation,
phase change, and microstructure kinetics of phase change. III. J. Chem. Phys..

[39] Avrami M. (1940). Kinetics of
phase change. II transformation-time relations for random distribution
of nuclei. J. Chem. Phys..

[40] Avrami M. (1939). Kinetics of
phase change. I General theory. J. Chem. Phys..

