# Supplementary material for: Chemical and Thermal Stability of Sr1.9VMoO6−δ: Implications for High Temperature Energy Conversion Applications
Source: ACS Omega. 2026 Feb 2;11(6):9050–8. doi: 10.1021/acsomega.5c06796 (PMC12917653; doi:10.1021/acsomega.5c06796)
Supplement: Supplementary file 1 [file ao5c06796_si_001.pdf]

Chemical and Thermal Stability of $Sr_{1.9}VMoO_{6-\delta}$: Implications for High Temperature Energy Conversion Applications

Bamidele J. Samuel[1], Julia A. Esakoff [2,3], Stephen K. Heywood[2,3], Stephen W. Sofie[2,3], Robert A. Walker[1,3*]

[1]Department of Chemistry and Biochemistry, Montana State University, Bozeman, MT 59717

[2]Department of Mechanical and Industrial Engineering, Montana State University, Bozeman, MT 59717

[3]Montana Materials Science Program, Montana State University, Bozeman, MT 59717

Supporting Information

Summary: 8 pages, 6 figures.

SI Figure 1: *In Situ* Raman spectra of $Sr_{1.9}VMoO_{6-\delta}$ in N_2 from room temperature to 1000 °C

SI Figure 2: *In Situ* Raman spectra of $Sr_{1.9}VMoO_{6-\delta}$ in 20% CO_2 from room temperature to 1000 °C

SI Figure 3: *In Situ* Raman spectra of $Sr_{1.9}VMoO_{6-\delta}$ in N_2 with 3% steam from room temperature to 1000 °C

SI Figure 4: Room-temperature powder XRD patterns of $Sr_{1.9}VMoO_{6-\delta}$: (a) as-fabricated, (b) after heating at 1000 °C in forming gas (5% H_2, 95% N_2), and (c) after heating at 1000 °C in air

SI Figure 5: Kinetic trace of $Sr_{1.9}VMoO_{6-\delta}$ degradation at 700 °C under REDOX gas conditions, with fits to double exponential and Avrami kinetic models

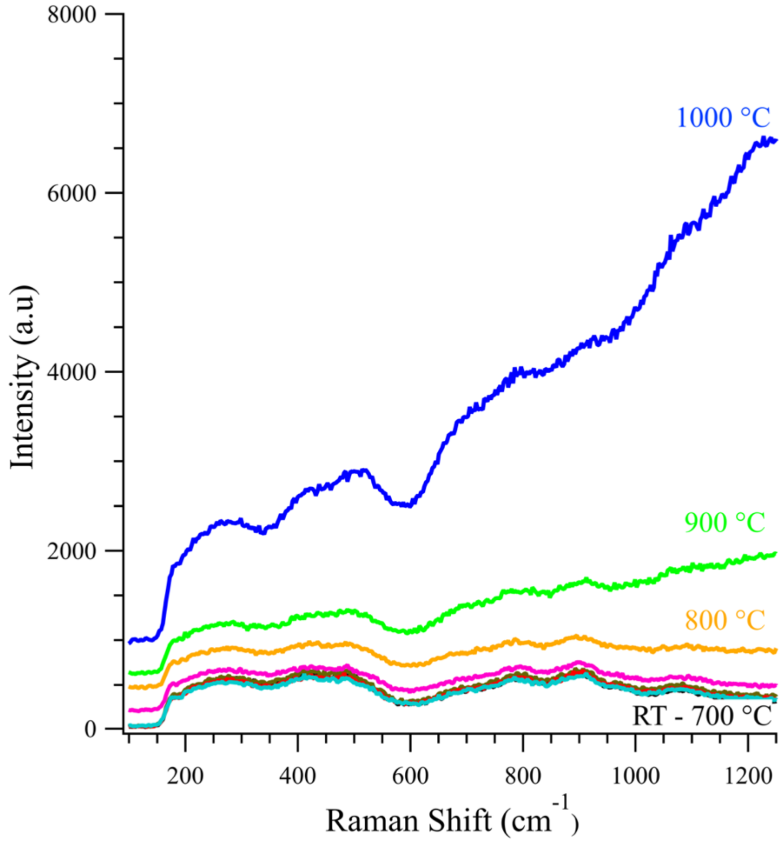

Figure S1: *In Situ* Raman spectra of $Sr_{1.9}VMoO_{6-\delta}$ from room temperature to 1000 °C under N_2.

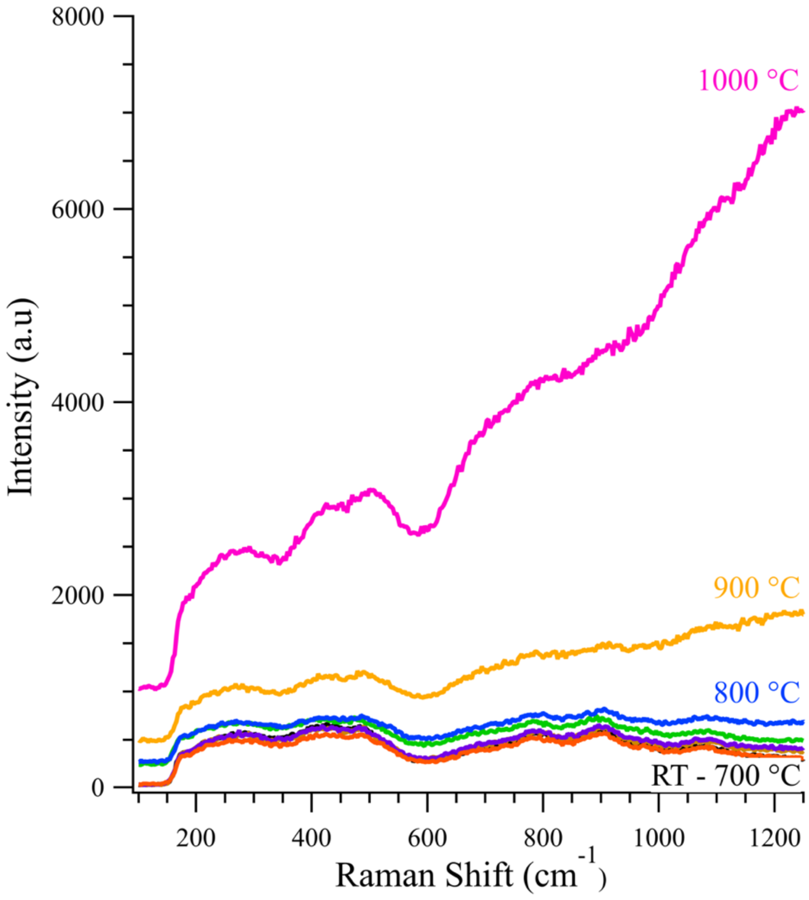

Figure S2: *In Situ* Raman spectra of $Sr_{1.9}VMoO_{6-\delta}$ from room temperature to 1000 °C under 20% CO_2/80% N_2.

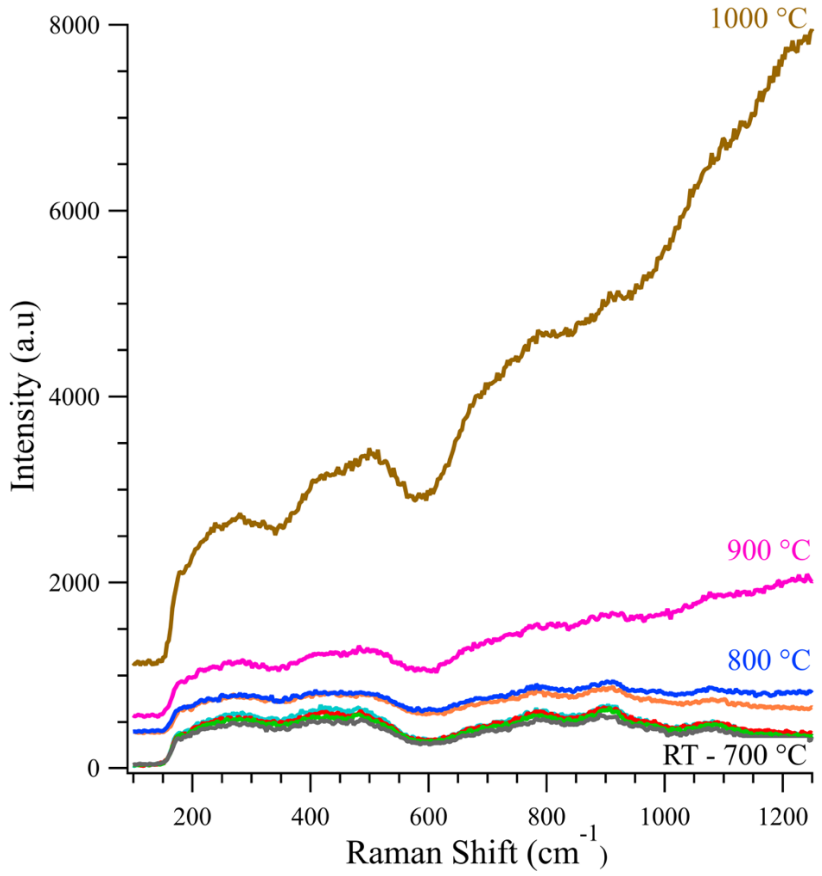

Figure S3: *In Situ* Raman spectra of $Sr_{1.9}VMoO_{6-\delta}$ from room temperature to 1000 °C in humidified N_2 containing~ 3% steam.

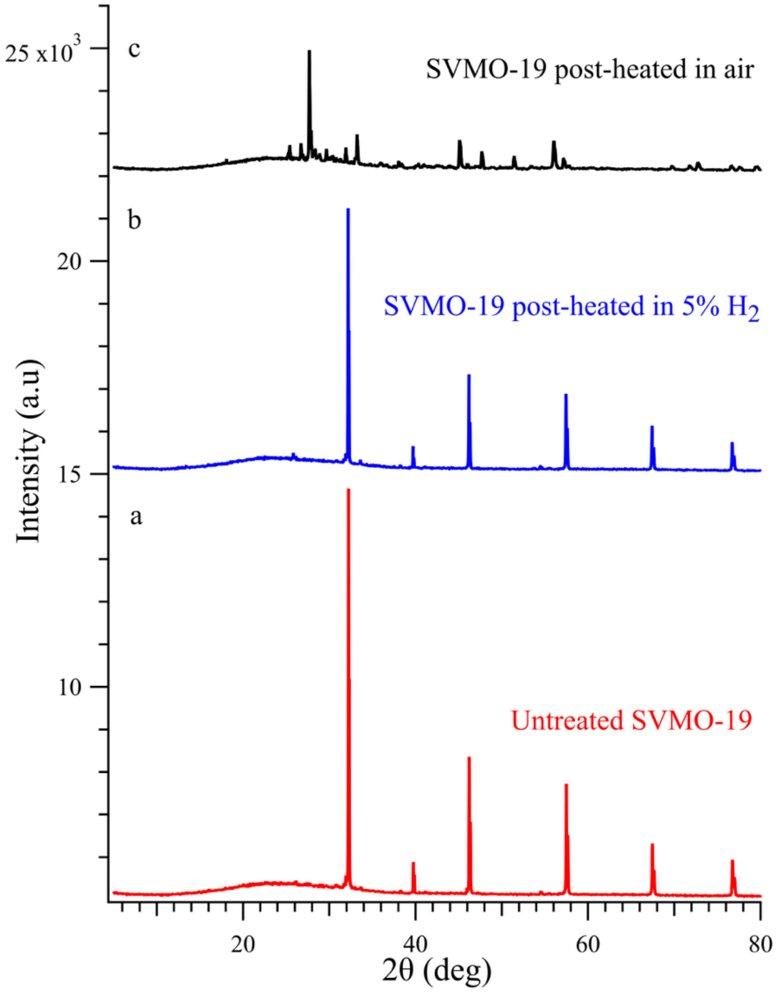

Figure S4: Room-temperature powder XRD patterns of $Sr_{1.9}VMoO_{6-\delta}$: (a) as-fabricated, (b) after heating at 1000 °C in forming gas (5% H_2, 95% N_2), and (c) after heating at 1000 °C in air. Space group for SVMO (Pm-3m)

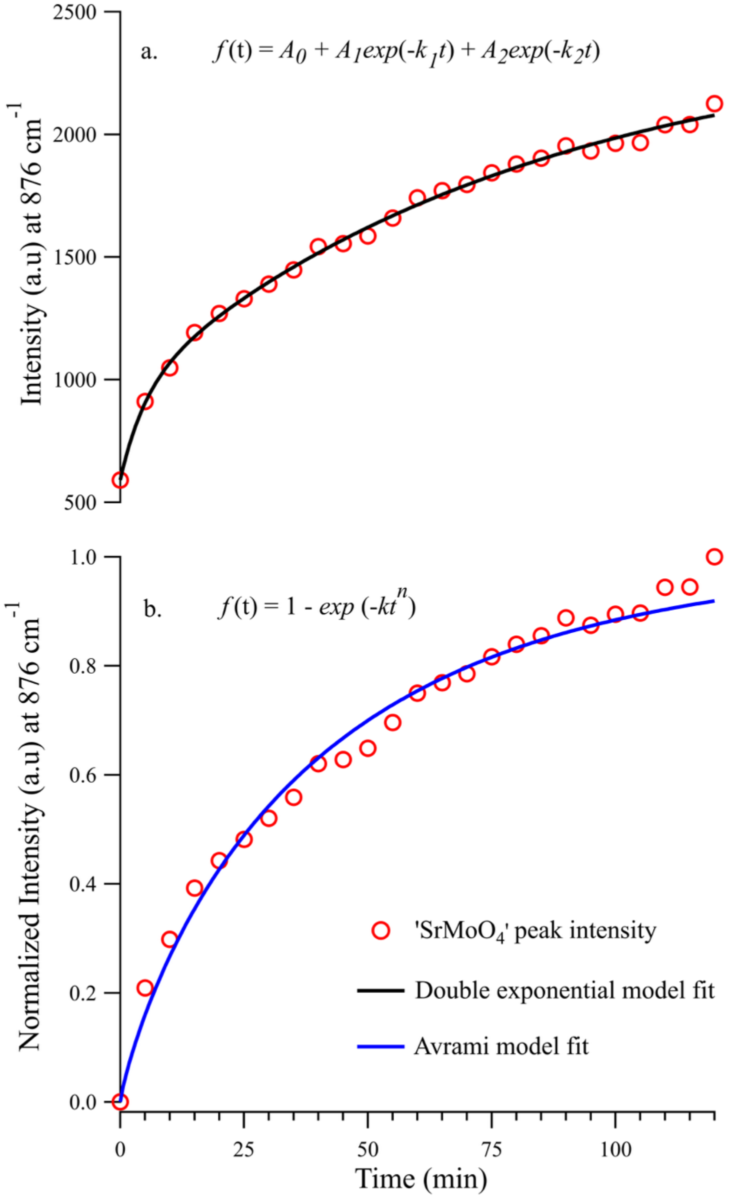

Figure S5: Kinetic trace of $Sr_{1.9}VMoO_{6-\delta}$ degradation at 700 °C taken under isothermal conditions. The sample is heated to 700˚C under forming gas, and then the atmosphere is switched to air. In air, SVMO-19 begins to decompose to form secondary phases including $SrMoO_4$. $SrMoO_4$ has a strong, sharp Raman feature at 876 cm^{-1}. The plots above show the growth of this feature (relative to baseline) after the atmosphere has been changed from reducing to oxidizing. The data are fit by both a double exponential function (top) and the Avrami model (bottom). See manuscript for more details.
